# ^18^F-fluoride PET/CT in clinical practice

**DOI:** 10.1590/0100-3984.2015.48.4e2

**Published:** 2015

**Authors:** Celso Darío Ramos

**Affiliations:** 1 PhD, Professor at School of Medical Sciences, Director, Service of Nuclear Medicine, Department of Radiology, Universidade Estadual de Campinas (Unicamp), Campinas, SP, Brazil. E-mail: cdramos@unicamp.com.br.

The presence of bone metastasis is a relevant prognostic factor for cancer patients, and the skeleton is a common site of distant metastasis in cases of advanced-stage neoplasias. Recent developments in oncological treatments have led to a significant increase in patients’ survival. On the other hand, much of the morbidity and disability in such patients is caused by metastatic bone disease. Thus an appropriate assessment of the skeleton for early detection of bone metastasis is essential for correct clinical decisions making in order to provide patients with appropriate treatment and better quality of life.

Bone scintigraphy (BS) with ^99m^Tc-labeled radiopharmaceuticals, such as ^99m^Tc-MDP, has been extensively utilized for the diagnosis of bone metastasis, and plays a relevant role in the initial assessment and follow-up of cancer patients. In spite of its relatively high sensitivity, the accuracy of BS is compromised by its limited spatial resolution^(1)^. The specificity is frequently low as the skeleton may be affected by different diseases with no specific scintigraphic findings^(2)^. For this reason, about one third of BSs present inconclusive results for bone metastasis, which implies the necessity of additional procedures for a definite diagnosis or for anatomical correlation, namely radiography, computed tomography, magnetic resonance imaging and biopsy^(3,4)^. The routine use of tomographic images such as SPECT/CT (single photon emission computed tomography/computed tomography) is also helpfull, but it significantly increases imaging acquisition time, particularly in cases where several segments of the skeleton must be scanned.

Positron emission tomography/computed tomography (PET/CT) with the radiopharmaceutical sodium fluoride labeled with fluorine18 (^18^F-NaF) has been utilized in the assessment of bone metastasis in a variety of malignancies^(4-6)^. The fast and intense ^18^F-fluoride uptake by active osteoblastic lesions and by the osteoblastic component of osteolytic lesions occurs due ^18^F-fluoride ions exchange with the hydroxyl groups in hydroxyapatite crystals. The method accuracy is quite high and several studies demonstrate that ^18^F-fluoride PET/CT is more sensitive and specific than BS to identify bone metastases due its higher spatial resolution and to the more favorable biokinetics of the radiopharmaceutical^(4-6)^.

In Brazil the availability of ^18^F-fluoride is limited, in spite of being produced for many years by Instituto de Pesquisas Energéticas e Nucleares (Ipen). Except for Ipen, all the other ten centers involved in the production of ^18^F-fluorodeoxyglucose (^18^F-FDG) do not commercialize ^18^F-fluoride, despite the simplicity of its production. Certainly, the main reason for that is the low demand for ^18^F-fluoride PET/CT scan since it is not covered by both public and private health care systems. In fact, the cost-effectiveness of ^18^Ffluoride PET/CT is still to be established, particularly in Brazil. The cost of the radiopharmaceutical ^18^F-fluoride is higher than that of MDP-^99m^Tc utilized in BS and, in addition, the operational cost of a PET/CT procedure is also higher than that of a scintillation camera. In this context, the time spent to perform a BS or a ^18^F-NaF PET/CT becomes one of the main factors that could balance the overall costs involved in the performance of each of the two procedures. Such an issue is the core of an extensive and definitive study developed by Ordones et al. and published in the previous issue of **Radiologia Brasileira**^(7)^.

The authors sought to establish the possibility of excluding lower limbs images from whole-body ^18^F-fluoride PET/CT scans as a means to reduce imaging acquisition time by 25% in the investigation of bone metastases. As much as one thousand whole-body ^18^F-fluoride PET/CT scans were reviewed by the authors and only three cases of bone metastases exclusively in the lower limbs were observed; and one of such lesions was not a true bone metastasis, rather, it was a soft tissue metastasis infiltrating adjacent bone tissue. Thus, the prevalence of this type of lesion corresponded to only 0.2%. Also, it is important to highlight that the two mentioned true exclusive lower limb metastasis found by the authors would have been detected at images acquired up to the root of the thighs, which has already been traditionally done and accepted for many years worldwide for most indications of ^18^F-FDG PET/CT scans^(8)^.

Generally, a BS is obtained three hours after radiopharmaceutical administration, and whole-body and additional static images acquisition takes about 30 minutes to be completed. As a SPECT or SPECT/CT acquisition is added, the patient remains in the examination room for at least additional 15 minutes. As necessary, further correlation with radiography, CT or magnetic resonance imaging is also performed, thus generating additional time and costs. On the other hand, a ^18^F-fluoride PET/CT scan can be initiated 30-60 minutes after radiopharmaceutical administration. An acquisition up to the proximal femurs as proposed by Ordones et al.^(7)^ does not take more than 12 minutes to be completed. Therefore, it might take less than one hour between ^18^F-fluoride injection and the patient discharge after image acquisition. It is possible to perform PET/CT scans in up to four patients per hour, which corresponds to at least twice the number of BSs performed during an equal period of time, particularly in cases where SPECT images are acquired. The acquisition of additional images, frequently needed in BSs, is almost never necessary in a ^18^F-fluoride PET/CT scan, and the procedure is completed with all the possible correlations with CT already performed – at least for anatomical correlation -, which considerably reduces the necessity of other imaging procedures. As noted by the authors, time savings also provide radiopharmaceutical savings, since ^18^F half-life is < 2 hours (in Brazil, multiple doses are provided in a single vial and they are locally fractionated in the PET/CT department, so as more scans can be performed with the activity of a single vial, provided each scanning time is shortened).

Currently, ^18^F-fluoride PET/CT is considered to be the most comprehensive imaging modality to evaluate metastatic bone disease^(9)^. The inclusion of such imaging method in the oncological routine depends on the establishment of practical and effective protocols whose costs are acceptable by the health care system. Therefore, it is clear the relevance of the study developed by Ordones et al. as a support for the incorporation of ^18^F-fluoride PET/CT imaging into the clinical practice.
